# Peroxisome proliferator-activated receptor-α-mediated transcription of *miR-301a* and *miR-454* and their host gene SKA2 regulates endothelin-1 and PAI-1 expression in sickle cell disease

**DOI:** 10.1042/BSR20150190

**Published:** 2015-12-08

**Authors:** Caryn S. Gonsalves, Chen Li, Punam Malik, Stanley M. Tahara, Vijay K. Kalra

**Affiliations:** * Department of Biochemistry and Molecular Biology, Keck School of Medicine of University of Southern California, Los Angeles, CA 90033, U.S.A.; † Department of Molecular Microbiology and Immunology, Keck School of Medicine of University of Southern California, Los Angeles, CA 90033, U.S.A.; ‡ Division of Experimental Hematology and Cancer Biology, Cincinnati Children's Hospital Medical Center, Cincinnati, OH 45229, U.S.A.

**Keywords:** endothelin-1 (ET-1), micro ribonucleic acid (miRNA), peroxisome proliferator-activated receptor-α (PPAR-α), plasminogen activator inhibitor-1 (PAI-1), sickle cell disease (SCD), spindle and kinetochore-associated protein-2 (SKA2)

## Abstract

Endothelin-1 (ET-1) and plasminogen activator inhibitor-1 (PAI-1) play important roles in pulmonary hypertension (PH) in sickle cell disease (SCD). Our previous studies show higher levels of placenta growth factor (PlGF) in SCD correlate with increased plasma levels of ET-1, PAI-1, and other physiological markers of PH. PlGF-mediated ET-1 and PAI-1 expression occurs via activation of hypoxia-inducible factor-1α (HIF-1α). However, relatively little is understood regarding post-transcriptional regulation of PlGF-mediated expression of ET-1 and PAI-1. Herein, we show PlGF treatment of endothelial cells reduced levels of *miR-301a* and *miR-454* from basal levels. In addition, both miRNAs targeted the 3′-UTRs of ET-1 and PAI-1 mRNAs. These results were corroborated in the mouse model of SCD [Berkeley sickle mice (BK-SS)] and in SCD subjects. Plasma levels of *miR-454* in SCD subjects were significantly lower compared with unaffected controls, which correlated with higher plasma levels of both ET-1 and PAI-1. Moreover, lung tissues from BK-SS mice showed significantly reduced levels of pre-*miR-301a* and concomitantly higher levels of ET-1 and PAI-1. Furthermore, we show that *miR-301a*/*miR-454* located in the spindle and kinetochore-associated protein-2 (SKA2) transcription unit was co-transcriptionally regulated by both HIF-1α and peroxisome proliferator-activated receptor-α (PPAR-α) as demonstrated by SKA2 promoter mutational analysis and ChIP. Finally we show that fenofibrate, a PPAR-α agonist, increased the expression of *miR-301a*/*miR-454* and SKA2 in human microvascular endothelial cell line (HMEC) cells; the former were responsible for reduced expression of ET-1 and PAI-1. Our studies provide a potential therapeutic approach whereby fenofibrate-induced *miR-301a*/*miR-454* expression can ameliorate PH and lung fibrosis by reduction in ET-1 and PAI-1 levels in SCD.

## Abstract (precis)

*miR-301a*/miR-*454*, which targets the 3′-UTR of ET-1 and PAI-1, are located in the intron of the SKA2 gene. These miRNAs are transcriptionally regulated by PPAR-α. Fenofibrate, a PPAR-α agonist increases levels of *miR-301a*/miR-*454*, with potential for amelioration of pulmonary hypertension.

## INTRODUCTION

Pulmonary disease, both acute and chronic, is the second most common cause of hospitalization and a leading cause of both morbidity and mortality in adults with sickle cell disease (SCD) [1,2]. Factors implicated in pulmonary hypertension (PH) in SCD include endothelial dysfunction, pulmonary vasoconstriction and vascular remodelling [3–6].

Endothelin-1 (ET-1) and nitric oxide (NO) are opposing pulmonary vasoactive factors that regulate pulmonary vascular tone [4,6–8]. Studies have shown that there is a clinical syndrome of haemolysis-associated PH in SCD [4]. Haemolysis leads to NO quenching by free haemoglobin in plasma, thereby reducing the bioavailability of this NO-synthase product [1,5,9]. ET-1 is a potent pulmonary vasoconstrictor and its levels are elevated in SCD patients with PH [10]. ET-1 is normally induced in endothelial cells in response to hypoxic activation of hypoxia-inducible factor-1 (HIF-1) [11,12]. Abrogation of this linkage by use of ET-1 receptor antagonists has been shown to be beneficial for treatment of primary PH [13]. This treatment has been found to be beneficial for PH treatment in sickle-Antilles-haemoglobin D mice [13], thus indicating the important role of ET-1 in PH in SCD. Other studies show that SCD patients have elevated steady state plasma levels of circulating tissue factor [14] and plasminogen activator inhibitor-1 (PAI-1), both of which increase further during sickle vaso-occlusive crises [15]. The prothrombic state thus predisposes patients to PH and stroke [16,17]. In addition to the role of PAI-1 in coagulation, it is responsible for development of lung injury/fibrosis; its expression is also modulated by other physiological stimuli such as hypoxia, transforming growth factor-β (TGF-β) and lipopolysaccharide (LPS) [18–20].

We have previously reported high circulating levels of placenta growth factor (PlGF) in SCD, compared with healthy control subjects, correlates with increased incidence of vaso-occlusive crises [21]. We also demonstrated the specific contribution of elevated PlGF to PH by ectopic, erythroid PlGF expression from a transduced, lentivirus vector, in normal mice to abnormally high levels also seen in Berkeley-SS mice [6]. These mice showed increased production of ET-1 with increased right ventricular pressures and right ventricle hypertrophy, both markers of PH [6]. These findings were corroborated in patients (*n*=123) with SCD, in whom plasma PlGF levels were associated with anaemia, an increase in ET-1 levels and increased tricuspid regurgitant velocity (TRV); the latter a marker of peak pulmonary artery pressure [6]. Studies by Brittain et al. [22] of 74 SCD patients found a positive correlation between high PlGF levels, haemolysis and inflammation. Moreover, we showed PlGF increased the expression of ET-1 and PAI-1 in human pulmonary microvascular endothelial cells (HPMVEC) and monocytes via activation of HIF-1α, independently of hypoxia [8,23]. Indeed, PlGF-mediated induction of PAI-1 was achieved by a transcriptional mechanism involving HIF-1α [23] and post-transcriptionally by specific miRNAs (*miR-30c* and *miR-301a*) that bind to the 3′-UTR of PAI-1 mRNA [24]. Our recent studies show *miR-199a2* targets the 3′-UTR of HIF-1α mRNA and concomitantly attenuates expression of HIF-1α and its downstream target genes, e.g. ET-1 [25].

In the present study, we examined the role of miRNAs in the post-transcriptional regulation of ET-1 and PAI-1. Our studies showed that *miR-301a* and *miR-454*, target the 3′-UTRs of ET-1 and PAI-1 mRNAs. A physiological relationship between *miR-301a* and *miR-454* was demonstrated *in vitro*, as both miRNAs attenuated PlGF-mediated secretion, in human microvascular endothelial cell line (HMEC-1), of both ET-1 and PAI-1. Moreover, these miRNAs co-located in an intron of the spindle and kinetochore-associated protein-2 (SKA2) gene were co-transcriptionally regulated by peroxisome proliferator-activated receptor-α (PPAR-α) and HIF-1α. The participation of PPAR-α in SKA2 transcription was corroborated by employing the PPAR-α agonist, fenofibrate. Furthermore, the expression of pre-*miR-301a* was significantly reduced in lung tissues harvested from sickle mouse model [Berkeley sickle mice (BK-SS)] animals compared with C57BK/6NJ controls. A similar relationship was observed in the plasma levels of *miR-454* of sickle cell anaemia (SCA) patients compared with healthy matched controls, where elevated ET-1 and PAI-1 levels are observed. The present study, to the best of our knowledge, is the first demonstration that PPAR-α co-regulates the transcription of SKA2, *miR-301a* and *miR-454*; the ET-1 mRNA has complementary sites in the 3′-UTR for the seed sequences of these miRNAs.

## MATERIALS AND METHODS

### Cell culture and reagents

HPMVECs, at passage two and growth media were obtained from Cell Applications Inc. These cells were cultured in growth media containing 10% FBS on 1% gelatin-coated tissue culture flasks or dishes according to manufacturer's instructions. These primary cells maintained characteristics of endothelial cell morphology and cell phenotype up to passage 6–7 and thus were not used beyond the 7th passage [26]. Immortalized HMEC-1, grown in culture, were not used beyond 20 passages [26]. These were cultured in RPMI-1640, supplemented with 10% FBS, sodium heparin, HEPES buffer, sodium pyruvate, non-essential amino acids and penicillin–streptomycin. Unless otherwise indicated, endothelial cells were cultured overnight in complete medium containing 2% serum, followed by incubation for 3 h in serum-free medium (SFM) prior to treatment with either PlGF (250 ng/ml) or other experimental conditions as indicated.

Recombinant human PlGF (Peprotech), primary antibodies against HIF-1α, ET-1, PAI-1, PPAR-α and secondary antibodies conjugated to horseradish-peroxidase (HRP) were obtained from Santa Cruz Biotechnology. Antibodies against β-actin were obtained from Sigma Chemical Company. Hsa-miR mimics and hsa-miR-inhibitors were purchased from Shanghai Gene Pharma Co. Ltd and Applied Biosystems. shRNAs for PPAR-α were generously provided by Dr Jae Jung (USC). siRNA for HIF-1α was obtained from Santa Cruz Biotechnology.

### Human subjects

All blood samples were obtained from children with homozygous SCA at steady state during their elective clinic appointments with routine clinical blood draws provided through the Hematology Repository at Cincinnati Children's Hospital Medical Center, Cincinnati, OH. All samples were obtained with the informed consent of the patient/legal guardian using Institutional Review Board approved-protocols at Cincinnati Children's Hospital. The plasma samples were obtained from the SCA patients and unaffected sibling as controls. Blood samples were analysed from patients who had no acute sickle events, fevers or infections 3 weeks before or 3 weeks after the blood draw, and were not transfused within the last 90 days.

### Animal studies

Animal protocols were approved by the Institutional Animal Care and Use Committee at Cincinnati Children's Hospital Medical Center. BK-SS originally obtained from Jackson Laboratories were bred up to the 6th generation against a C57BL/6NJ background. The BK-SS mice carry deletions for the mouse α- and β-globin genes, and carry a transgene expressing the human α-globin and a mini β-globin cluster, including the β-sickle globin as described [25,27]. Six male BK-SS mice at 10–12 weeks of age were used for these experiments. Five female and one male, in-house bred C57BL/6NJ animals (10–12 weeks old) served as controls. Mice were exsanguinated and lungs were removed for analysis; all tissues were stored at −80°C and later assayed for miRNAs, ET-1 and PAI-1.

### Isolation of RNA and expression of mRNA and miRNA by qRT-PCR

Endothelial cells were treated with PlGF for indicated time periods followed by total RNA extraction using TriZOL reagent (Invitrogen) as per manufacturer's protocol. mRNA and pre-miRNA expression were determined and quantified using specific primers (Table 1). Quantitative real-time PCR (qRT-PCR) of mRNA and pre-miRNA templates was performed using the iScript One-Step RT-PCR Kit with SYBR Green detection (Bio-Rad) in an ABI Prism 7900 HT sequence detection system (Applied Biosystems, Foster City, CA). Briefly, PCR amplification of 100 ng of total RNA was performed for 40 cycles under the following conditions: cDNA synthesis at 42°C for 10 min, iScript reverse transcriptase inactivation at 95°C for 10 s and PCR cycling and detection at 95°C for 5 s, followed by 60°C for 34 s. Values are expressed as relative expression of mRNAs or pre-miRNAs, normalized to endogenous GAPDH mRNA levels [24,28]. Mature miRNAs were isolated using mirVana isolation kit (Applied Biosystems) and miRNA expression levels were determined using the TaqMan MicroRNA Assay Kits for indicated miRNAs (Applied Biosystems), as previously described [24].

**Table 1:** Oligonucleotide primers used in the present study Abbreviations: qRT-PCR, quantitative real-time PCR; SDM, site directed mutagenesis.

Gene	Method	Forward primer	Reverse primer
SKA2 mRNA	qRT-PCR	GTTCCAGAAAGCTGAGTCTGA	TTGCTGAATCAGGATGATTAGTCT
GAPDH mRNA	qRT-PCR	AACCTGCCAAGTACGATGACATC	GTAGCCCAGGATGCCCTTGA
Pre-*miR-301a* RNA	qRT-PCR	CTGCTAACGAATGCTCTGAC	CCTGCTTTCAGATGCTTTGAC
Pre-*miR-454* RNA	qRT-PCR	GATCCTAGAACCCTATCAATATTG	CCCATTGTTCTTTCCAAACACC
mPAI-1	qRT-PCR	GTA TGA CGT CGT GGA ACT GC	TTTCTCAAAGGGTGC AGC GA
mET-1	qRT-PCR	TGCCTCTGAAGTTAGCCGTG	AGTTCTCCGCCGCCTTTTTA
mGAPDH	qRT-PCR	TTGCAGTGGCAAAGTGGAGA	GTCTCGCTCCTGGAAGATGG
mpre*miR-301a*	qRT-PCR	GCTAACGGCTGCTCTGACTT	CTCGCGGATGCTTTGACAAT
SKA2 HRE1M (−193/−189)	SDM	TTGTGATTGGCTGAGATagatcAGAGCTC- CAGCCAATCAG	CTGATTGGCTGGAGCTCTgatcTATCTCA- GCCAATCACAA
SKA2 HRE2M (−223/−219)	SDM	CGGATGGTGTAGGAgatcAGCCCGGCTTG- TGA	TCACAAGCCGGGCTgatcTCCTACACCAT- CCG
SKA2 del PPAR-α	SDM	GATATGAAGAAGTAAAAACCTCCCAAG- AGAATTTGACAATATTAGAATATATTCC	GGAATATATTCTAATATTGTCAAATTC- TCTTGGGAGGTTTTTACTTCTTCATATC
SKA2 promoter (PPRE at −1671/−1653)	ChIP	GCTAAGTGTTAGCTAGCAAGTGT	GCCAACTCCTGCAAAAGACT
ET-1 *miR-301a*/*454* site 1 mutant	SDM	TGGCCGACTCcatcCTCTCCACCCTGG	CAGGGCTCTCCGTGGAGG
ET-1 *miR-301a*/*454* site 2 mutant	SDM	TCACCTATATcatcCTCTGGCAGAAGTATTTC	GGTAGACTCATATTCATGAAAC
PAI-1 *miR-301a*/*454* site 1 mutant	SDM	ATGGATGTAAcatcCTTTGGGAGGCCAAGGC	CTTTGTGCCCTACCCTCTG
PAI-1 *miR-301a*/*454* site 2 mutant	SDM	TTTTTGATTTcatcCTGGACGGTGACG	AGAAAGAAAGAAAAACCCCAAAG

### Generation of SKA2 promoter luciferase constructs and 3′-UTR reporter luciferase constructs for ET-1 and PAI-1

The SKA2 promoter luciferase construct was generated using the Infusion Cloning kit. Briefly, the 5′-flanking region of SKA2 spanning nts −2000 to +12 was PCR amplified from human BAC clone RP11-626H11 (BACPAC Resources Center) with the Phusion PCR kit (New England Biolabs), and amplified product was inserted into the pGL3-Basic vector. The 3′-UTR for ET-1 was PCR amplified from human BAC clone RP11-353G10 and inserted into the unique XbaI site, 3′ to the reporter gene in the pGL3-Control vector. PAI-1 3′-UTRs were PCR amplified from BAC clone RP11-213E22 and inserted into the pMIR vector using the Infusion cloning kit (Clontech) and primers listed in Table 1. Deletions of the PPAR-α site and mutation of the HIF-1α-binding site, within the SKA2 promoter, mutations within the *miR-301a*/*miR-454*-binding sites in the ET-1 and PAI-1 3′-UTRs were all carried out using the Q5 site-directed mutagenesis kit (New England Biolabs) in the corresponding parental vectors using primers listed in Table 1. All constructs and mutations were verified by sequencing.

### Transient transfections

Transient transfections of endothelial cells were performed using the Amaxa Nucleofector [25]. Briefly, 1×10^6^ cells per transfection were collected by centrifugation and resuspended in 100 μl of RPMI-1640, along with the indicated plasmids. Cells were then electroporated using the Y-001 transfection protocol. Transfected cells were incubated for 24 h in complete media, followed by serum deprivation for 12 h prior to treatment with PlGF. For luciferase reporter experiments, 1 μg each of the luciferase plasmid and Renilla luciferase plasmid was used. For RNAi experiments, 1 μg of the shRNA plasmid, 90 pmol of the miRNA mimics and miRNA inhibitors, as indicated, were used for transfections.

### Luciferase assays

Luciferase assays were conducted using the Dual-luciferase assay kit (Promega), as per the manufacturer's protocol. Briefly, cells were centrifuged and lysed in 1× passive lysis buffer; 20 μl of the lysates were used for the luciferase assays. Luciferase values were normalized to Renilla luciferase values. Data are expressed relative to the parent vector or promoterless vector, as appropriate.

### Western blot analysis

Whole cell lysates were prepared from cells as previously described [25]. Extracts were subjected to electrophoresis on a 12% SDS/PAGE gel followed by electrophoretic transfer to a PVDF membrane. Membranes were probed for HIF-1α, ET-1 and PAI-1 proteins utilizing antibodies to HIF-1α (1:250), ET-1 (1:500) and PAI-1 (1:500), respectively. Blots were stripped and reprobed with a β-actin antibody to monitor protein loading. The immunoreactive protein bands were detected using the Clarity Western ECL Solution (Bio-Rad). The quantitation of band intensities was performed on scanned images utilizing ImageJ Software.

### ChIP assays

HMEC-1 cells in serum free media were treated with PlGF (250 ng/ml). ChIP analysis was performed using an antibody to HIF-1α and PPAR-α (Santa Cruz Biotechnology) as previously described [25]. DNA was subjected to PCR amplification for 30 cycles using the following conditions: 95°C for 45 s, 63°C for 30 s, 72°C for 120 s, using primers listed in Table 1. The PCR products were analysed by electrophoresis on a 2% agarose gel, visualized by ethidium bromide staining and quantified using the ImageJ analysis software [25].

### Quantification of secreted ET-1 and PAI-1

HMEC-1 (1×10^5^ cells) were transfected with indicated miRNA mimic or negative controls (NC mimic or inhibitors; 90 pmol) as described above and incubated in complete media at 37°C for 24 h. Cells were washed with SFM and incubated for 3 h in SFM (2 ml) treatments were begun by replacement with fresh SFM (1 ml) and additions, as indicated. Cells were treated with either PlGF (250 ng/ml) or fenofibrate (0.1 mM) overnight. The culture supernatant was collected and an aliquot (0.1 ml) was assayed for ET-1 (R&D Systems) and PAI-1 (Peprotech) release using an ELISA kit. The cells were collected by scraping and the pellet was assayed for protein content utilizing the Bradford method.

### Statistical analysis

Data are presented as means±S.D. One-way ANOVA, followed by Tukey–Kramer test were used for multiple comparisons using the Instat-2 software (Graph Pad). *P* values of less than 0.05 were considered significant.

## RESULTS

### *miR-301a* and *miR-454*, located in the first intron of host gene SKA2, are co-transcriptionally regulated by PPAR-α and HIF-1α

Our previous studies show PlGF treatment of HPMVECs attenuates expression of *miR-30c* and *miR-301a*, which target the 3′-UTR of PAI-1 mRNA and thus affect its turnover [24]. *In silic*o analysis showed *miR-301a* is located in the first intron of the SKA2 gene and is co-localized with *miR-454* as shown in the gene schematic (Figure 1A). Further analysis predicted that *miR-454* also could interact with the 3′-UTRs of ET-1 and PAI-1. We began by examining the time course of expression of SKA2, pre-*miR-301a* and pre-*miR-454* mRNA by qRT PCR, in response to PlGF in HMEC-1. We observed that PlGF treatment of HMEC resulted in a time-dependent increase in SKA2 mRNA expression with maximal increase of ∼10-fold at 4 h (Figure 1B). The expression of pre-*miR-301a* and pre-*miR-454* mRNA showed a maximal increase in ∼4-fold at 2 h, followed by a gradual decline after 4 h to almost basal level by 8 h (Figure 1B). Moreover, PlGF-mediated SKA2 expression was attenuated by shRNA for phosphoinositide 3-kinase (PI3K), shRNAs for mitogen-activated protein kinase (MAP kinase) and c-Jun (Figure 1C), indicating the roles of PI3K, MAP kinase and c-Jun in the transcription of SKA2. Furthermore, these results indicated that pri-miRNA synthesis and pre-miRNA processing preceded SKA2 transcription and splicing, as expected from the 5′-proximal location of the miRNA genes within SKA2. Alternatively an independent promoter for pri-miRNA transcription could be operative. In an effort to distinguish between these two possibilities further analysis of SKA2 and miRNA transcription was performed.

**Figure 1:**
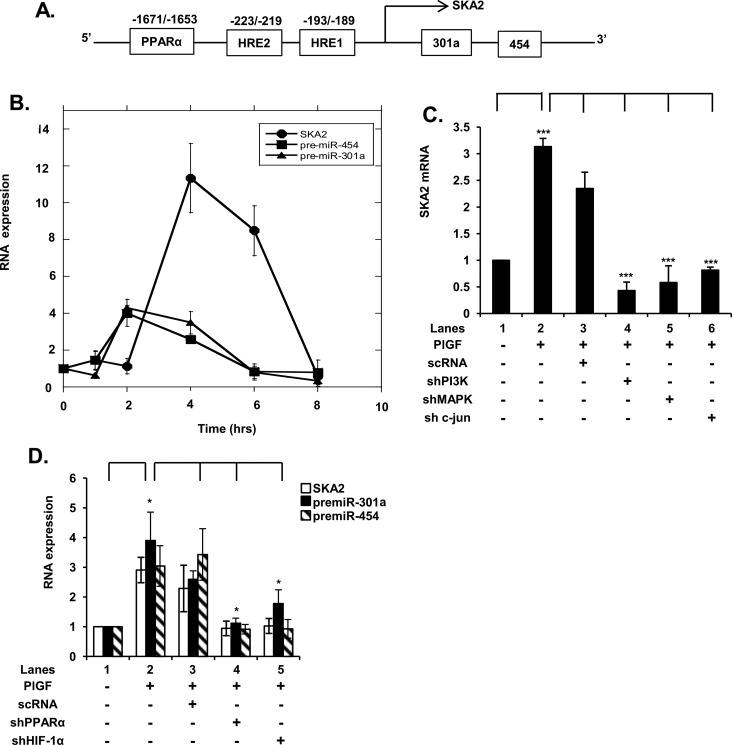
PlGF up-regulates the expression of *miR-301a* and *miR-454* located in an intron of host gene SKA2 by activation of HIF-1α and PPAR-α (**A**) Schematic of 5′ end of SKA2 gene showing locations of *miR-301a* and *miR-454* in the first intron of SKA2 and positions of *cis*-binding elements for HIF-1α and PPAR-α. (**B**) Time-dependent (1–8 h) expression of SKA2, pre-*miR-301a* and pre-*miR-454* RNA. (**C**) Effect of transfection of shRNAs for PI3K, MAPK and c-Jun on SKA2 mRNA expression. HMEC cells were transfected with shRNAs for 24 h, followed by treatment with PlGF for 4 h. (**D**) Effect of transfection of shRNAs for HIF-1α and PPAR-α on PlGF-mediated SKA2 transcription following 2 h incubation. Data are means±S.D. of three independent experiments. ***P<0.001, **P<0.01, *P<0.05, ^ns^*P*>0.05.

*In silico* analysis of the 5′-flanking ∼2 kb region of SKA2 revealed the presence of *cis*-binding elements for PPAR-α and binding sites for HIF-1α (HRE, hypoxia response element), as shown in Figure 1(A). The presence of these transcription factor-binding sites within the SKA2 promoter guided our decision to perform a gene knockdown approach. As shown in Figure 1(D), transfection of HMEC with PPAR-α shRNA and HIF-1α shRNA attenuated PlGF-mediated induction of SKA2 mRNA to the basal level (Figure 1D, lanes 4 and 5 compared with lane 2). Moreover, the knockdown of PPAR-α and HIF-1α also reduced >90% the expression of pre-*miR-301a* and pre-*miR-454* (Figure 1D). Taken together these data showed that pre-*miR-301a* and pre-*miR-454* were co-transcribed with the SKA2 primary transcript, induced by PlGF, and were not products of a secondary transcription unit within the SKA2 intron.

### PlGF-mediated transcription of SKA2 involves PPAR-α as demonstrated by PPAR-α agonist, promoter analysis and ChIP

In order to further extend our observation regarding the participation of PPAR-α in SKA2 transcription, we employed the synthetic PPAR-α agonist fenofibrate and the PPAR-α antagonist GW6471 for their effects on SKA2 transcription. Treatment of HMEC with fenofibrate resulted in a ∼3-fold increase in SKA2 mRNA, pre-*miR-301a* and pre-*miR-454*. Moreover, the induction of SKA2 mRNA, pre-*miR-301a* and pre-*miR-454* was reduced by >80% by GW6471 (Figure 2A). In addition, fenofibrate addition also augmented by 3-fold the expression of mature *miR-301a* and *miR-454*, both of which were attenuated by GW6471 (Figure 2B). These results clearly showed by a functional approach that synthesis of the latter miRNAs was linked to the initiation of transcription of SKA2.

**Figure 2:**
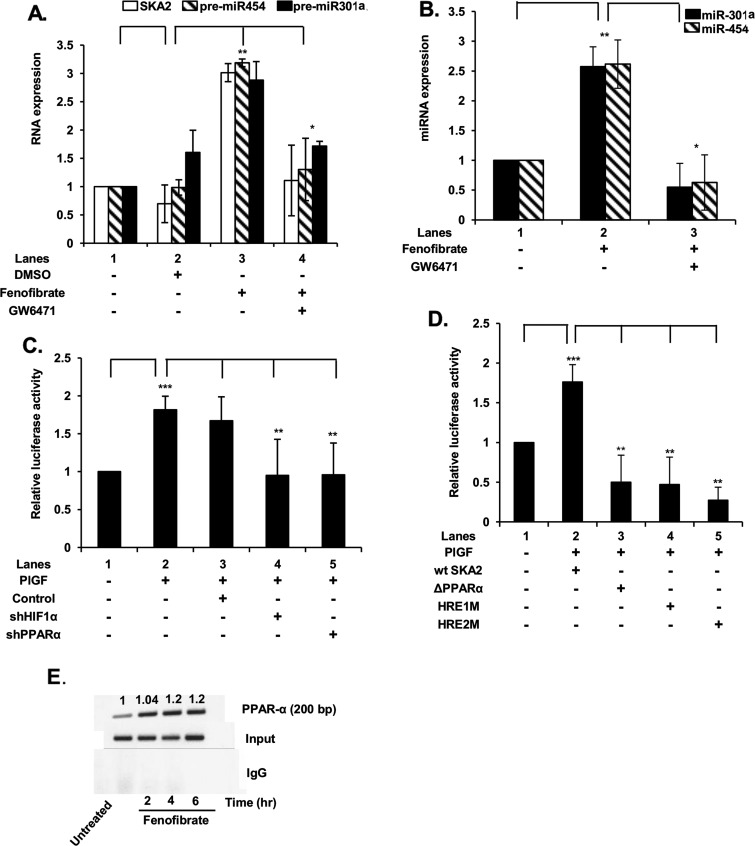
PlGF-mediated expression of SKA2 transcription unit is regulated by PPAR-α and HIF-1α as demonstrated by SKA2 promoter analysis and ChIP (**A**) Effect of PPAR-α agonist (fenofibrate) and its antagonist (GW6471) on SKA2, pre-*miR-301a* and pre-*miR-454* RNA expression. HMEC cells were pre-treated with GW6471 (15 μM) for 30 min, where indicated, followed by treatment with fenofibrate (100 μM) for 2 h. (**B**) Effect of fenofibrate on induction of mature *miR-301a* and *miR-454*. (**C**) Effect of shRNA for HIF-1α and PPAR-α on PlGF-mediated transcription of SKA2, as demonstrated utilizing wt SKA2 promoter-luciferase reporter. HMEC were co-transfected overnight with wt SKA2-luc and indicated shRNAs along with Renilla luciferase plasmid, followed by treatment with PlGF for 4 h. Cell lysates were assayed for luciferase activity and data normalized for Renilla luciferase activity. (**D**) Effect of mutation of HRE sites and deletion of PPAR-α sites in wt SKA2 promoter of SKA2-luc reporter. HMEC cells were transfected with the wt SKA2 promoter-luc or SKA2-luc reporters with mutations in either HRE site 1 or HRE site 2 or deletion of PPAR-α *cis*-binding element. Cells were co-transfected with Renilla luciferase plasmid for 24 h and were treated with PlGF for 4 h. Luciferase activity was normalized to Renilla luciferase activity to correct for transfection efficiency and the data are expressed as relative expression compared with the luciferase activity of wt construct, as indicated in the figure. Data are means±S.D. of three independent experiments. ***P<0.001, **P<0.01, *P<0.05, ^ns^*P*>0.05. (**E**) ChIP analysis of HMEC cells treated with fenofibrate for 2–6 h, for assay of PPAR-α binding to the SKA2 promoter. PPAR-α antibody (top row) or control rabbit IgG (bottom row) were used for immunoprecipitation of soluble chromatin. The middle panel represents the amplification of input DNA before immunoprecipitation. Densitometric analysis, showing relative intensity of PPAR-α PCR product normalized to input DNA are indicated above each lane. Data are representative of two independent experiments.

Since PlGF induced SKA2 mRNA expression in HMEC, we utilized a SKA2 promoter luciferase reporter gene construct to further delineate the roles of PPAR-α and HIF-1α in this promoter. The wt SKA2-luc plasmid (∼2 kb) has provisionally identified PPAR-α and HIF-1α *cis*-binding promoter elements as depicted in the schematic diagram in Figure 1(A). HMEC transfected with the wt SKA2 luc reporter plasmid followed by treatment with PlGF showed a ∼1.8-fold increase in luciferase activity (Figure 2C, lane 2 compared with lane 1). The promoter construct was functionally the same as the endogenous promoter as shown by the effect of shRNA for PPAR-α and HIF-1α, both of which attenuated PlGF induced luciferase activity (Figure 2C).

Next, we determined *cis*-binding element regions for PPAR-α in the SKA2 promoter, which were responsible for SKA2 transcription. HMEC were transfected with the wt promoter (∼2000 bp) luciferase reporter and a promoter construct deleting the PPAR-α site located at nts −1671 to −1673. Expression of this PPAR-α deleted SKA2 promoter in HMEC, following PlGF treatment resulted in reduction in luciferase activity below the basal level when compared with the intact wt SKA2 promoter (Figure 2D, lane 3 compared with lane 2). Similarly, mutation of either HRE-1 or HRE-2 sites in the SKA2 promoter completely abrogated PlGF induction of luciferase activity (Figure 2D, lanes 4 and 5 compared with lane 2). These results confirmed the predicted transcription factor utilization in the initiation of SKA2 transcription, showing that both PPAR-α and HIF-1α were actively involved in transcription.

An assessment of binding of PPAR-α to the SKA2 promoter was confirmed by analysis of native chromatin in HMEC using the ChIP assay. For this analysis, we utilized chromatin from HMEC cells treated with a PPAR-α agonist, fenofibrate. HMEC were incubated with fenofibrate up to 8 h followed by ChIP with PPAR-α antibody or control rabbit IgG. Fenofibrate treatment of HMEC showed ∼3-fold increase in an expected 200 bp PCR product that corresponds to nt −1736 to −1537 of the SKA2 promoter region containing the PPAR-α site (Figure 2E). The recovery of the expected PCR product reached a maximum after 4 h and remained constant through 8 h, post fenofibrate addition (Figure 2E). As shown, amplification of the input DNA prior to immunoprecipitation was equivalent in all the samples (Figure 2E, middle panel) and the absence of signal in the control IgG immunoprecipitate confirmed the specificity of the anti-PPAR-α immunoprecipitation. The combination of these promoter studies clearly established that PPAR-α participates in SKA2 transcription.

### *miR-301a* and *miR-454* target 3′-UTR of ET-1 and attenuate ET-1 mRNA and protein levels

*In silico* analysis of the 3′-UTR of ET-1 mRNA predicted *miR-301a*/*miR-454* miRNA recognition element (MRE) sequences at nucleotides +45/+65 and +572/+593, as depicted in the schematic of Figure 3(A)**.** Although the 3′ complementary regions of these miRNAs are different, the 5′-domains of each are capable of base pairing to the same MRE in the ET-1 3′-UTR. We tested this prediction by examining the effects of synthetic *miR-301a* and *miR-454* on PlGF induced ET-1 mRNA expression in both HMEC and HPMVEC. As shown in Figure 3B, transfection with both *miR-301a* and *miR-454* mimics reduced PlGF-mediated ET-1 mRNA to basal levels. We next examined the role of the ET-1 3′-UTR as the sole determinant of *miR-301a* and *miR-454* targeting in a luciferase translation reporter, in response to PlGF. Upon co-transfection of pGL3-ET-1 3′-UTR-luc with exogenous *miR-301a* or *miR-454* mimics, we observed ∼90% decrease in luciferase activity, compared with PlGF treated cells (Figure 3C, lanes 4 and 5 compared with lane 2). These data showed that both *miR-301a* and *miR-454* target the 3′-UTR of ET-1 mRNA to attenuate ET-1 synthesis.

**Figure 3:**
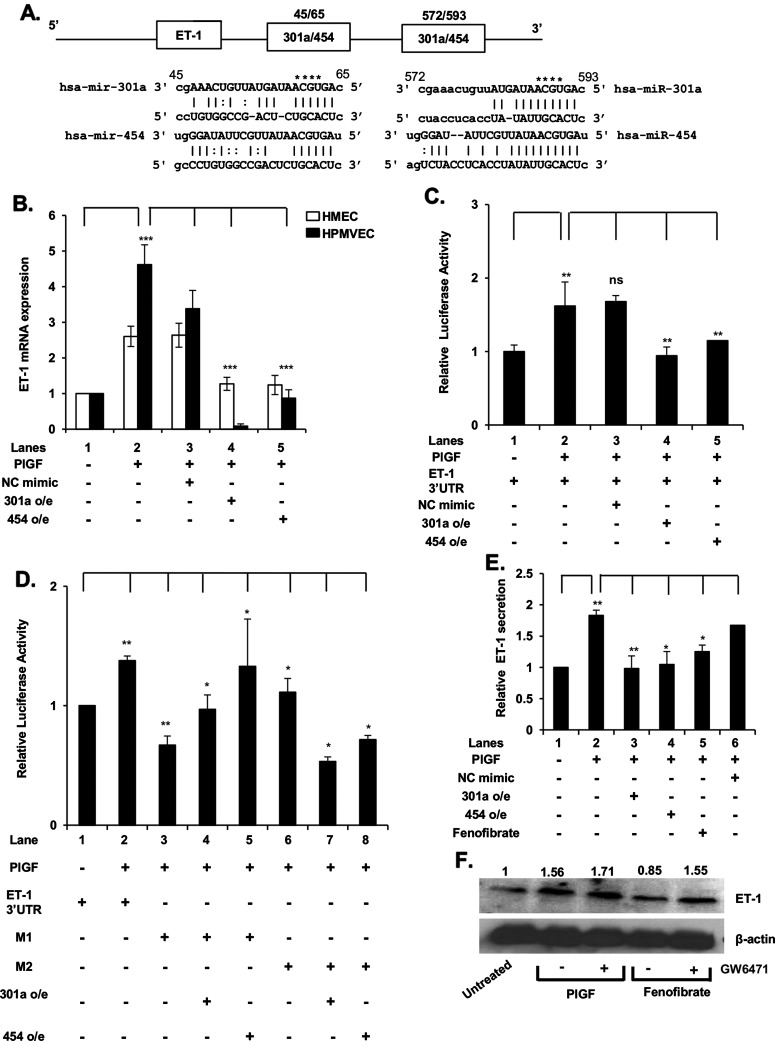
*miR-301a* and *miR-454* target the 3′-UTR of ET-1 mRNA (**A**) Schematic of 3′-UTR of ET-1 mRNA showing locations of MRE for *miR-301a* and *miR-454*. (**B**) Effects of *miR-301a* and *miR-454* synthetic mimics on PlGF-mediated ET-1 mRNA expression. HMEC and HPMVEC cells were transfected with *miR-301a* mimic (90 pmol), *miR-454* mimic (90 pmol) or control (NC mimic) overnight, followed by treatment with PlGF for 6 h. (**C**) Effect of *miR-301a* and *miR-454* synthetic mimics on 3′-UTR-ET-1-luciferase activity. HMEC cells were transfected with wt pGL3-3′-UTR-ET-1 luc reporter along with indicated miRNA mimics, followed by PlGF treatment for 6 h. (**D**) Effect of mutation of MRE sites for *miR-301a*/*miR-454* in wt 3′-UTR-ET-1 luc. The sequences of predicted *miR-301a*-/*miR-454*-binding sites within ET-1 3′-UTR are shown in panel A. The bases in bold font indicate seed sequences of *miR-301a*/*miR-454* and the base substitutions created in the corresponding MRE sequences are indicated by asterisks in panel A. HMEC were transfected with wild type ET-1 3′-UTR-luc or the indicated ET-1 3′-UTR mutant constructs (M1 or M2) along with either synthetic *miR-301a* or *miR-454*. At 24 h post-transfection, cells were treated with PlGF for 6 h. Cells were lysed in reporter assay buffer for assay of luciferase activity and data were normalized for Renilla activity. Results shown are means±S.D. of three independent experiments. ***P<0.001, **P<0.01, *P<0.05, ^ns^*P*>0.05. (**E**) Effect of synthetic *miR-301a* and *miR-454* mimics on PlGF-induced secretion of ET-1 from HMEC. HMEC were transfected with synthetic miR mimics (90 pmol). At 24 h post-transfection, mimic treated and control cells were treated with either PlGF or fenofibrate for 24 h, as indicated. The cell culture media were assayed for secreted ET-1 by ELISA and total protein content of cell lysates was determined. The data are expressed as relative release of ET-1 from HMEC cells. Data are means±S.D. of three independent experiments. (**F**) Effects of PPAR-α agonist (fenofibrate) and antagonist (GW6471) on ET-1 protein expression in HMEC cells. Densitometric analysis showing relative density values of ET-1 protein normalized to β-actin protein as a loading control, are indicated above each lane. Data are representative of two independent experiments.

In order to clearly establish the relationship between the putative miRNA recognition sites in the ET-1 3′-UTR and *miR-301a* and *miR-454*, we mutated both MREs for *miR-301a*/*miR-454* in pGL3-ET-1 3′-UTR-luc. As shown in Figure 3(D), PlGF augmented luciferase activity in the reporter plasmid containing the wt ET-1 3′-UTR (Figure 3D, lane 2 compared with lane 1). However, mutation of the MRE site denoted M1 (nt +60/+63) of the ET-1 3′-UTR (shown in Figure 3A), prevented the inhibitory effects of exogenous *miR-301a* and *miR-454* on luciferase activity. Similarly, mutation of the MRE site denoted M2 (nt +584/+588) of the ET-1 3′-UTR, resulted in attenuation of PlGF-induced luciferase activity (Figure 3D, lanes 7 and 8 compared with lane 6). These data showed that both MREs in the 3′-UTR of ET-1 mRNA were required for functional effects of *miR-301a* and *miR-454*.

To determine whether *miR-301a* and *miR-454* reduced ET-1 protein synthesis via interaction with the 3′-UTR of ET-1 mRNA, we measured the secretion of ET-1 protein from HMEC cells. As shown in Figure 3(E), PlGF treatment of HMEC resulted in ∼2-fold increase in the secretion of ET-1 protein. Transfection of HMEC with exogenous *miR-301a* or *miR-454* mimic reduced PlGF-mediated ET-1 secretion to the basal level (Figure 3E, lanes 3 and 4 compared with lane 2).

As shown above, fenofibrate increased the expression of *miR-301a* and *miR-454* by inducing transcription of SKA2, accordingly we examined its effect on ET-1 secretion. Fenofibrate treatment of HMEC reduced PlGF-induced ET-1 secretion by ∼80% (Figure 3E). The effect of fenofibrate on ET-1 synthesis was due to reduced mRNA translation as demonstrated by Western blot. The effect of fenofibrate on ET-1 synthesis was reversed by GW6471, a PPAR-α antagonist (Figure 3F). Taken together these data showed that increased levels of *miR-301a* and *miR-454* decreased expression of ET-1, induced by PlGF.

### *miR-301a* and *miR-454* target the 3′-UTR of PAI-1 and effect a decrease in PAI-1 mRNA and protein levels

*In silico* analysis of the 3′-UTR of PAI-1 mRNA showed the presence of *miR-301a*/*miR-454* recognition elements at nt +1351/+1373 and at nt +1641/+1663 as depicted in the schematic of Figure 4(A)**.** Thus, we examined the effects of exogenous *miR-301a* and *miR-454* on PlGF-induced PAI-1 mRNA levels in both HMEC and HPMVEC. As shown in Figure 4(B), transfection with *miR-301a* and *miR-454* mimics in HMEC and HPMVEC reduced PlGF-induced PAI-1 mRNA to basal level and below basal level, respectively. Transfection with control NC mimic had no effect on PlGF-mediated PAI-1 mRNA levels (Figure 4B, lane 3 compared with lane 2). Next, we examined binding of *miR-301a* and *miR-454* to their target sequences in the PAI-1 3′-UTR of pGL3-PAI-1-3′-UTR reporter in response to PlGF. Upon co-transfection of pGL3-PAI-1-3′-UTR- with exogenous *miR-301a* or *miR-454* mimics, there was ∼90% decrease in luciferase activity, compared with PlGF-treated cells (Figure 4C, lanes 4 and 5 compared with lane 2). These data showed that both *miR-301a* and *miR-454* target the 3′-UTR of PAI-1 mRNA to attenuate PAI-1 translation.

**Figure 4:**
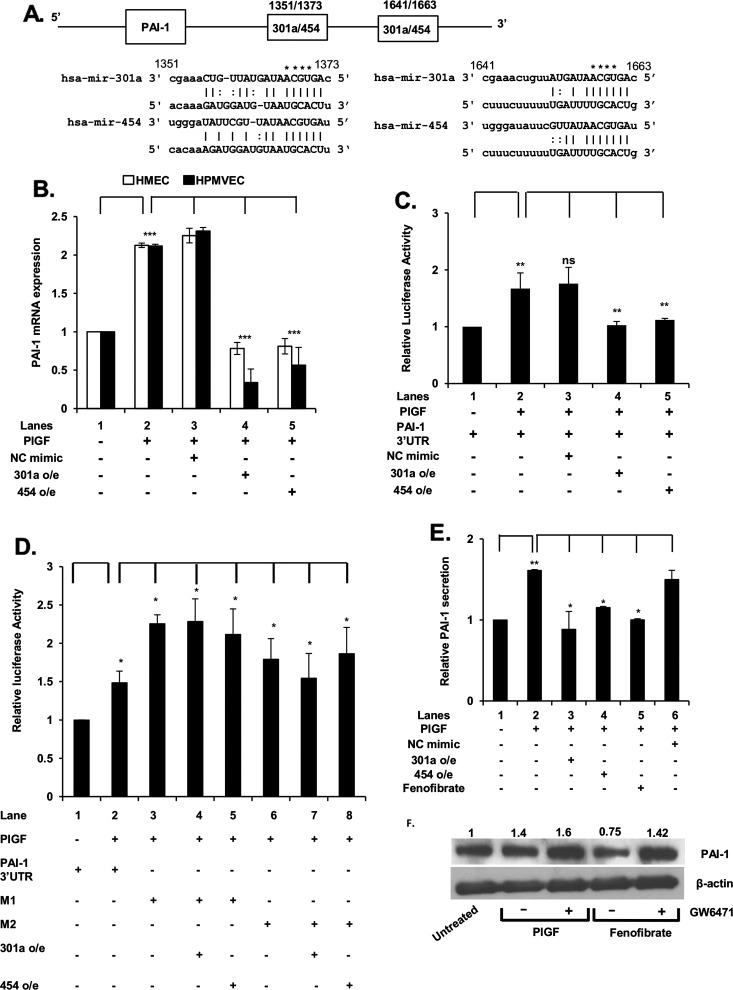
*miR-301a* and *miR-454* target the 3′-UTR of PAI-1 mRNA (**A**) Schematic of 3′-UTR of PAI-1 mRNA showing locations of MRE for *miR-301a* and *miR-454*. (**B**) Effect of *miR-301a* and *miR-454* synthetic mimics on PlGF-mediated PAI-1 mRNA expression. HMEC and HPMVEC cells were transfected with synthetic *miR-301a* mimic (90 pmol), *miR-454* mimic (90 pmol) or control (NC mimic) as described in Figure 3 legend. (**C**) Effect of *miR-301a* and *miR-454* synthetic mimics on 3′-UTR-PAI-1-luciferase activity. (**D**) Effect of mutation of MRE sites for *miR-301a*/*miR-454* in wt 3′-UTR-PAI-1 luc. The sequences of predicted *miR-301a*-/*miR-454*-binding sites within PAI-1 3′-UTR are shown. The bases in bold font indicate seed sequences of *miR-301a*/*miR-454* and the base substitutions in the MRE sequences are indicated by asterisks in panel A. HMEC were transfected with wild type PAI-1 3′-UTR-luc or the indicated PAI-1 3′-UTR mutant constructs (M1 or M2) along with either *miR-301a* or *miR-454*. Cells were lysed in reporter assay buffer for assay of luciferase activity and data are normalized for Renilla activity. Results are means±S.D. of three independent experiments. ***P<0.001, **P<0.01, *P<0.05, ^ns^*P*>0.05. (**E**) Effect of synthetic *miR-301a* and *miR-454* mimics on PlGF-induced secretion of PAI-1 from HMEC. The data are expressed as relative secretion of PAI-1 from HMEC cells. Results are means±S.D. of three independent experiments. (**F**) Effects of PPAR-α agonist (fenofibrate) and antagonist (GW6471) on PAI-1 protein expression in HMEC cells. Densitometric analysis of PAI-1 protein expression with values normalized to β-actin as a loading control are indicated above each lane. Data are representative of two independent experiments.

Next, we examined whether mutation of MRE sites for *miR-301a*/*miR-454* in pGL3-PAI-3′-UTR affected reporter activity with exogenous *miR-301a* and *miR-454*. As shown in Figure 4(D), exogenous PlGF augmented wt PAI-1 3′-UTR reporter luciferase activity (Figure 3D, lane 2 compared with lane 1). However, mutation of the MRE sites located at nt +1367/+1370 (M1) and at positions +1657/+1660 (M2) of the PAI-1 3′-UTR (see schematic in Figure 4A) in the luciferase reporter gene, prevented either exogenous *miR-301a* or *miR-454* from negatively affecting luciferase activity (Figure 4D). These data showed that both MRE sites were effective functional targets in the 3′-UTR of PAI-1 mRNA for *miR-301a* and *miR-454*.

We extended our study of miRNA effects on PAI-1 synthesis to determine whether *miR-301a* and *miR-454* affected PlGF-induced PAI-1 protein expression, as reflected by changes in PAI-1 secretion from HMEC. As shown in Figure 4(E), PlGF treatment of HMEC resulted in ∼1.8-fold increase in the secretion of PAI-1 protein. Transfection of HMEC with exogenous *miR-301a* or *miR-454* mimic attenuated PlGF-mediated PAI-1 protein release to the basal level (Figure 4E, lanes 3 and 4 compared with lane 2).

In an effort to induce endogenous *miR-301a* and *miR-454*, fenofibrate treatment of HMEC augmented the endogenous expression of *miR-301a* and *miR-454* and subsequently reduced the secretion of PlGF-induced, endogenous PAI-1 by ∼90% (Figure 4E). This effect was not due to inhibition of secretion but was indeed due to decreased PAI-1 synthesis as shown by Western blot analysis of whole cell lysates. Fenofibrate attenuated PAI-1 protein expression whereas GW6471, a PPAR-α antagonist, reversed the effect of fenofibrate on PAI-1 protein synthesis (Figure 4F). The sum of these studies showed that *miR-301a* and *miR-454* could be induced *in situ* by fenofibrate to antagonize PlGF-induction of PAI-1 synthesis and secretion. The effect of these miRNAs was elaborated through their interaction with the 3′-UTR of PAI-1 mRNA.

### *miR-454* expression *in vivo*

We previously demonstrated *miR-301a* levels are depressed in SCD subjects compared with matching controls [24]. As we show in this report, *miR-454* is also functional as a post-transcriptional regulator of PAI-1 synthesis and co-synthesized with *miR-301a*, consequently it was necessary to establish that *miR-454* levels paralleled *miR-301a* in both normal and in SCD.

We examined *miR-301a* expression in lungs from BK-SS sickle mice and control C57BL/6NJ mice. Mice do not have an orthologue for human *miR-454*, although the location of *miR-301a* is syntenic between human and mouse. We previously showed that *miR-301a* is depressed in plasma from BK-SS mice [24]. We extended this observation by examining the expression of PAI-1 and *miR-301a* in mouse lung. As shown in Figure 5(A), *miR-301a* expression in lung tissue from BK-SS mice compared with control mice was not significantly different. As a model for PH, the BK-SS mice also expressed higher levels of ET-1 and PAI-1 mRNAs and proteins as shown in Figures 5(A) and 5(B), respectively. These results were further extended in human subjects, wherein *miR-454* in plasma of SCD patients was also reduced significantly in comparison with levels determined in control subjects (Figure 5C). These results extended our previous observation that plasma from SCD patients showed decreased expression of *miR-301a* with correspondingly higher levels of PAI-1. Furthermore, these results demonstrated that *miR-301a* and *miR-454* are indeed co-regulated in SCD and undergo down regulation by an as yet unidentified process. Thus co-regulated reduction in *miR-301a* and *miR-454* appears to significantly contribute to the observed increase in ET-1 and PAI-1 seen in SCD.

**Figure 5:**
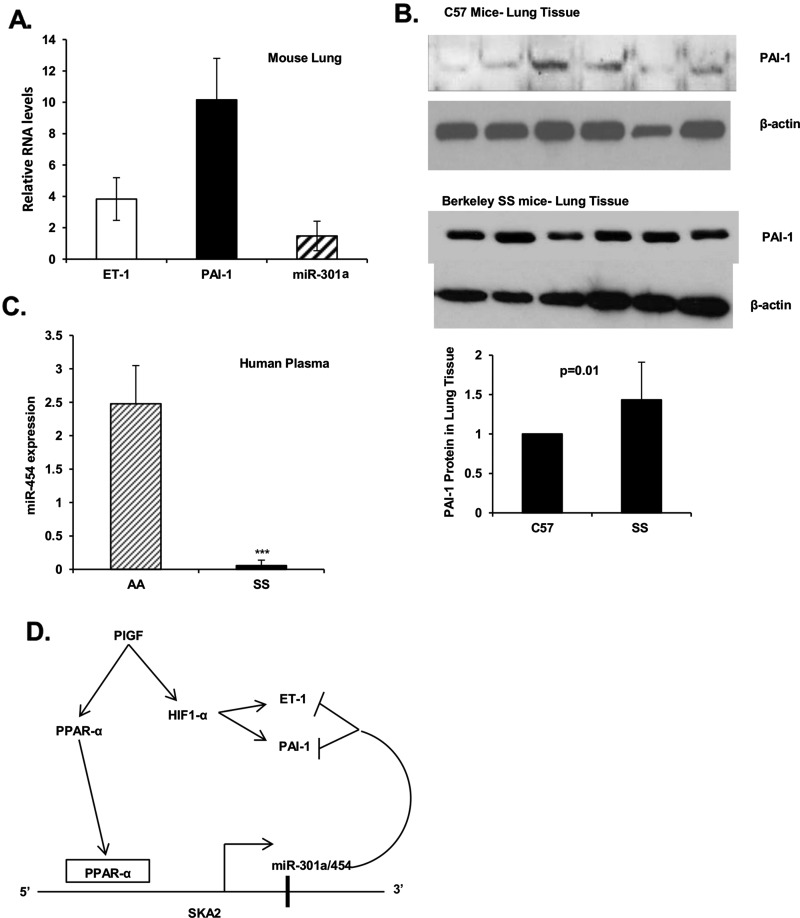
Expression of *miR-301a* and *miR-454* in lung tissues of BK-SS and SCD human subjects (**A** and **B**) Frozen lung tissue samples (−80°C storage) from BK-SS mice (*n*=6) and control C57BL/6NJ mice (*n*=6) were sliced and homogenized directly in RNA extraction buffer or protein cell lysate buffer. (**A**) Extracted RNA was analysed for ET-1 and PAI-1 mRNA expression by qRT-PCR utilizing primers indicated in Table 1. The data were normalized to GAPDH as internal control. miRNA was isolated from lung tissues using miRNA isolation kit, followed by PCR analysis. miRNA levels were normalized to internal reference 5S rRNA. The data are expressed as relative RNA levels in BK-SS mice compared with control mice. (**B**) Western blots of cell lysates of lung tissue from BK-SS and control C57BL/6NJ mice utilizing ET-1 and PAI-1 antibodies. Gel lanes correspond to individual animals. Lower panel, combined densitometric quantitation of PAI-1 expression in sickle (BK-SS) compared with control animals (C57). (**C**) *miR-454* expression in plasma of SCD subjects compared with sibling controls (*n*=10). (**D**) Working model of PlGF induced transcription of SKA2 transcription unit, encompassing intronic *miR-301a*/*miR-454*, by activation of PPAR-α and binding to the 5′-flanking region of the SKA 2 promoter. *miR-301a*/*miR-454* target the 3′-UTR of ET-1 mRNA and the 3′-UTR of PAI-1 mRNAs leading to reduction in corresponding ET-1 and PAI-1 levels. The reduced expression of *miR-454* in SCD will lead to increased ET-1 and PAI-1 expression.

## DISCUSSION

Our previous studies show higher circulatory levels of PlGF in the BK-SS model and SCD patients are associated with increased levels of plasma ET-1 and other markers of PH, including PAI-1 [6]. We previously showed that expression of ET-1 and PAI-1 was transcriptionally regulated by HIF-1α, independent of hypoxia [8,23]. Our studies also show PlGF attenuates the expression of *miR-30c* and *miR-301a* in cultured endothelial cells, both of which target the 3′-UTR of PAI-1 under basal conditions [24]. Although extensive biochemical reagents and computational tools based on public data sets are available for identification of specific mRNA gene targets by miRNAs, there is a dearth of information describing transcriptional regulation of miRNA genes in the scheme of post-transcriptional regulation of gene expression [29–31].

The transcription factor Twist-1 regulates expression of the *miR-199a2*/*miR-214* cluster during development of specific neural cell populations in mouse embryogenesis [32]. Recently, we showed this miRNA cluster is also transcriptionally regulated by PPAR-α [25]. *miR-199a2* targets the 3′-UTR of HIF-1α mRNA; increased expression of the former reduces transcription of hypoxia and stress-related genes. We demonstrated that PPAR-α agonists induced transcription of the *miR-199a2*/*miR-214* cluster leading to down-regulation of HIF-1α levels, manifested by significant decreases in HIF-1α-dependent gene transcription. Together this work clearly demonstrated that this miRNA cluster is subject to differential regulation in response to changes in physiological conditions.

In the present study, we extended our prior report regarding the regulatory activity of *miR-301a* on PAI-1. The transcriptional regulation of *miR-301a* was previously examined in the context of PAI-1 synthesis. Additionally a re-analysis of the PAI-1 mRNA showed two additional *miR-301a* sites in its 3′-UTR. The search algorithm used previously (Microcosm: http://www.ebi.ac.uk/enright-srv/microcosm/cgi-bin/targets/v5/search.pl) predicted the site at nt +422 to +444 [24], but not the *miR-301a* sites at nt+1351/+1373 and nt +1641/+1663. The latter sites have complementarity to the seed region of *miR-301a* and were also identified as potential *miR-454*-binding sites (www.microrna.org). Thus *miR-301a* and *miR-454* comprised a miRNA set which potentially interacted with PAI-1 mRNA. This became of interest because both *miR-301a* and *miR-454* are co-synthesized from the SKA2 transcription unit.

The functional consequence of binding *miR-301a* and *miR-454* to target gene mRNAs ET-1 and PAI-1 was demonstrated by use of synthetic *miR-301a* and *miR-454* mimics. Both miRNAs attenuated PlGF-induced ET-1 and PAI-1 expression in HMEC. The specificity of the interaction between *miR-301a* and *miR-454* and their respective MREs in the 3′-UTR of ET-1 mRNA was established by inclusion of the ET-1 3′-UTR in a luciferase translation reporter. This reporter responded to synthetic *miR-301a* and *miR-454* mimics like endogenous ET-1 mRNA. Mutation of the *miR-301a*- and *miR-454*-binding sites in the luciferase translation reporter confirmed the functional interaction between these miRNAs and the 3′-UTR of ET-1 mRNA.

The first intron of SKA2 gene includes genes for *miR-301a* and *miR-454*. Ska2 participates in mitotic spindle formation and function during metaphase; it is also important for monitoring aberrant kinetochore assembly during the spindle check point step of mitosis [33]. Although the miRNA genes are in the correct polarity to be part of the SKA2 transcription unit, it was still conceivable that a smaller transcription unit within the intron was responsible for *miR-301a*/*miR-454* synthesis. Our studies showed PlGF coordinately induced the expression of pre-*miR-301a*, pre-*miR-454* and SKA2 mRNA, which was maximal at 4 h followed by complete return to basal level by 8 h. These expression kinetics would be expected for normal expression of this locus from a common promoter. The importance of this level of post-transcriptional regulation is consistent with our previous demonstration that PlGF leads to a reduction in *miR-301a* and *miR-454* expression, thus providing a permissive cell state enhancing the PlGF induction of ET-1 and PAI-1 expression in SCD [6,23].

As further validation of miRNA coexpression with SKA2, we showed that *cis*-binding elements for PPAR-α and HIF-1α associated with the SKA2 promoter were functionally important for PlGF-induced transcription of the SKA2 gene. This was demonstrated using shRNAs specific for PPAR-α and HIF-1α, which antagonized production of SKA2 mRNA as well as both *miR-301a* and *miR-454*. The SKA2 promoter was further examined in a luciferase reporter construct where we showed mutation of the HRE and deletion of the PPAR-α *cis*-elements rendered the promoter insensitive to induction by PlGF. These transcription factor *cis*-elements were found to be functional for SKA2 in native chromatin as demonstrated by ChIP assay probes specific for the factor-binding sites in the SKA2 promoter.

The participation of PPAR-α in SKA2 transcription was of particular interest to us because PPAR-α agonists could be useful for induction of the latter gene. We found that fenofibrate treatment of HMEC induced the co-expression of SKA2, the precursors of *miR-301a* and *miR-454* and their mature forms. The effect of this agonist was specific since co-incubation of fenofibrate-treated cells with the PPAR-α antagonist GW6471 prevented induction as reflected by the absence of any increase in SKA2 or miRNA transcription.

The fenofibrate induction of SKA2 and *miR-301a*/*miR-454*, leading to attenuation of ET-1 expression in HMEC, may in fact also be assisted by fenofibrate-dependent reduction in HIF-1α expression. Our laboratory previously showed fenofibrate induces transcription of *miR-199a2* from the DNM3os locus [25]. The latter gene produces a long noncoding RNA, serving as pri-miRNA for *miR-199a2* and *miR-214*, the transcription of which is subject to trans-activation by PPAR-α. We demonstrated that *miR-199a2* targets the 3′-UTR of HIF-1α mRNA for down-regulation, leading to decreased transcription of the ET-1 and PAI-1 genes [25].

The data presented in the present study may explain the physiological effect of chronically high PlGF levels in SCD. PlGF transiently induced *miR-301a* and *miR-454* in HMEC after 4 h, however expression quickly returned to basal levels by 6 h. In other words, SKA2 transcription became refractory to PlGF induction after 6 h by an unknown process preventing sustained transcription of the SKA2 gene. Indeed, SCD patients exhibit abnormally high plasma levels of PlGF and were observed to express lower plasma levels of *miR-301a* and *miR-454*. This physiological phenomenon is also observed in the BK-SS model, which exhibits higher plasma levels of PlGF and abnormal levels of ET-1 with other functional markers of PH. Our studies showed that lung tissues from BK-SS mice displayed reduced levels of *miR-301a* consistent with the observation in human SCD patients. We propose that increased levels of ET-1 and PAI-1, as previously shown, [6] arises from the SKA2 gene becoming transcriptionally inactive upon chronic PlGF exposure. Loss of *miR-301a* and *miR-454* expression would therefore render ineffective a proposed feedback loop controlling ET-1 and PAI-1 levels (Figure 5D).

In conclusion, our studies provided further mechanistic insight into the co-regulation of *miR-301a* and *miR-454* and their relationship to ET-1 mRNA and its translation. The present study, to the best of our knowledge, is the first demonstration that PPAR-α co-regulates the transcription of SKA2, *miR-301a* and *miR-454*; the ET-1 mRNA has complementary sites in the 3′-UTR for the seed sequences of these miRNAs. Our studies on the PlGF responsiveness of SKA2 transcription may explain how chronic PlGF expression as observed in human SCD subjects and in the mouse SCD model results in abnormal expression of ET-1 and PAI-1. High level expression of the latter physiological mediators is a significant causal factor in PH [6,23]. These studies also revealed that fenofibrate, a PPAR-α agonist, approved by the Food and Drug Administration for the treatment of dyslipidemia [34] may increase the expression of both *miR-301a* and *miR-454* leading to decreased ET-1 and PAI-1. In fact, fenofibrate has a secondary effect of reducing HIF-1α levels by inducing transcription of *miR-199a2*, which post-transcriptionally suppresses HIF-1α synthesis [25]. Thus our studies provide a mechanistic basis for fenofibrate-mediated transcriptional regulation of *miR-301a* and *miR-454* in the present study and *miR-199a2* [25] as a rational therapeutic approach to attenuate elevated levels of ET-1 and PAI-1 observed in PH of SCD.

## Abbreviations

BK-SS: Berkeley sickle mice
ET-1: endothelin-1
HIF-1α: hypoxia-inducible factor-1α
HMEC: human microvascular endothelial cell line
HPMVEC: human pulmonary microvascular endothelial cells
HRE: hypoxia response element
MAP kinase: mitogen-activated protein kinase
MRE: miRNA recognition element
NC: negative control
NO: nitric oxide
PAI-1: plasminogen activator inhibitor-1
PH: pulmonary hypertension
PI3K: phosphoinositide 3-kinase
PlGF: placenta growth factor
PPAR-α: peroxisome proliferator-activated receptor-α
qRT-PCR: quantitative real-time PCR
SCA: sickle cell anaemia
SCD: sickle cell disease
SFM: serum-free medium
SKA2: spindle and kinetochore-associated protein-2
